# Patient-Reported Outcome Measures in Adults with Type 2 Diabetes—With a Focus on Older Populations: An AI-Assisted Rapid Review of Use and Implementation in Clinical and Organizational Practice

**DOI:** 10.3390/healthcare13222840

**Published:** 2025-11-08

**Authors:** Rossella Messina, Maria Pia Fantini, Michael Lodi, Paolo Di Bartolo, Rabih Chattat, Jacopo Lenzi

**Affiliations:** 1 Department of Biomedical and Neuromotor Sciences, University of Bologna, 40126 Bologna, Italy; jacopo.lenzi2@unibo.it; 2 Ravenna Diabetes Center, Department of Specialistic Medicine, Romagna Local Health Authority, 48100 Ravenna, Italy; paolo.dibartolo@auslromagna.it; 3 University of Bologna, 40126 Bologna, Italy; mariapia.fantini@unibo.it; 4 Department of Computer Science and Engineering, University of Bologna, 40126 Bologna, Italy; michael.lodi@unibo.it; 5 Department of Medical and Surgical Sciences, University of Bologna, 40126 Bologna, Italy; 6 Department of Psychology, University of Bologna, 40126 Bologna, Italy; rabih.chattat@unibo.it

**Keywords:** PROMs, type 2 diabetes, psychological aspects, aging, artificial intelligence

## Abstract

**Background/Objectives**: The aging global population has led to a rising prevalence of type 2 diabetes mellitus (T2DM), in which biomedical outcomes alone fail to capture patients’ lived experiences. Patient-Reported Outcome Measures (PROMs) can provide insights into psychological, psychosocial, and quality-of-life (QoL) dimensions, yet their use—particularly among older adults—remains inconsistent. This AI-assisted rapid review aimed to map how PROMs are currently applied in adults with T2DM, with specific attention to studies involving older populations, focusing on their role in assessing well-being, distress, depression, and treatment satisfaction, as well as their implementation in clinical and organizational practice. **Methods**: A rapid review was conducted using Elicit, an AI tool designed to support systematic evidence synthesis. Studies published between 2015 and 2025 were identified from Semantic Scholar, complemented by manual searches for recent or unindexed papers. Eligibility criteria required inclusion of adults with T2DM and use of validated PROMs in real-world settings. Studies explicitly describing older or elderly subgroups were highlighted separately. After screening 504 records, 167 studies were included. Data extraction covered study design, instruments used, populations, outcomes, and implementation details. **Results**: The most frequently assessed outcomes were diabetes distress, depression, QoL, treatment satisfaction, and self-efficacy. Common instruments included PAID, DDS, PHQ-9, WHO-5, EQ-5D, SF-36, DTSQ, and GDS. Evidence showed PROMs effectively identified high-risk patients and informed tailored interventions, but integration into routine care remained limited. Barriers included workflow disruption, lack of provider training, heterogeneity of tools, and insufficient cultural validation. Facilitators included brief instruments, digital administration, and linkage with care planning. **Conclusions**: PROMs are valuable in capturing psychosocial and psychological burdens in adults with T2DM, including but not limited to older populations, but routine implementation is inconsistent. Broader adoption will require digital infrastructure, clinician training, and organizational integration, as well as the development of PROMs that capture experiences with emerging diabetes technologies. Methodologically, this study illustrates the feasibility of AI-assisted rapid reviews to generate timely, evidence-informed syntheses.

## 1. Introduction

The global aging of the population has led to a marked increase in non-communicable diseases, with type 2 diabetes mellitus (T2DM) among the most prevalent. This demographic and epidemiological transition poses major challenges for healthcare systems, requiring a move beyond narrow biomedical targets toward more holistic, person-centered care. In older adults with T2DM, it is crucial to consider psychological well-being, functional status, and quality of life (QoL)—domains often affected by emotional distress, cognitive decline, and loss of independence but insufficiently captured by standard clinical assessments [1].

Although this review was designed with a focus on older adults, the available evidence rarely disaggregates outcomes by age. Many studies include broad adult populations, and only a subset explicitly targets participants aged ≥65 years. For this reason, the present review should be interpreted as an exploratory mapping of PROM use in adult T2DM care, highlighting where evidence is specific—or transferable—to older populations.

Patient-Reported Outcome Measures (PROMs) provide valuable insight into these dimensions by capturing patients’ own perspectives on symptoms, mood, and daily functioning, thereby informing personalized strategies and supporting shared decision-making. Yet their use in diabetes care remains inconsistent. Terwee et al. [2] underscored issues of heterogeneity, conceptual overlap, and poor standardization, all of which limit clinical utility. Barnard-Kelly and colleagues [2,3] emphasized the potential of PROMs to guide tailored psychological and behavioral interventions but also highlighted their underuse in routine practice. The same group further explored challenges specific to pediatric diabetes, where age-appropriate content and caregiver reporting complicate assessment [4].

Together, these studies underscore the need for more consistent integration of PROMs across patient groups and care contexts.

The International Consortium for Health Outcomes Measurement (ICHOM) [5] has proposed a core set of outcomes for adults with type 1 and T2DM—including PROMs such as the World Health Organization–Five Well-Being Index (WHO-5) [6,7], the Patient Health Questionnaire-9 (PHQ-9) for depressive symptoms [8], and the Problem Areas in Diabetes (PAID) scale for diabetes-related distress [9]—to be assessed at least annually. This reflects a growing consensus that psychological and QoL metrics should be monitored alongside traditional clinical outcomes. Nonetheless, real-world implementation remains limited by systemic barriers, including inadequate digital infrastructure, insufficient clinician training, and a lack of actionable pathways for PROM use in practice [10]. In parallel, the rapid spread of diabetes technologies—such as continuous glucose monitoring and insulin pumps—calls for PROMs that can capture patients’ digital and technology-related experiences, an aspect still largely unexplored.

To address these gaps, health systems increasingly require rapid and reliable evidence to guide both clinical and organizational choices. Timely, evidence-informed decision-making has therefore become a core requirement, particularly when action must be taken under significant time constraints and with clear accountability to patients and health systems. Rapid reviews have emerged as fit-for-purpose evidence products that retain core principles of systematic reviews while streamlining steps to deliver actionable syntheses in weeks rather than months [11,12,13]. In parallel, semi-automation and trustworthy artificial intelligence (AI) can accelerate several tasks, provided their use is coupled with human oversight and auditability. Evaluations have shown meaningful efficiency gains with machine-learning and crowdsourcing approaches, without unacceptable losses in accuracy when appropriately configured [14,15].

The performance of Elicit, an AI-based literature review assistant [16], was assessed by Bernard and colleagues [17], who compared its basic “find paper” functionality with a traditional umbrella review. Elicit retrieved 83% of the relevant papers identified manually; notably, conclusions remained unchanged when analyses were based only on the six studies found by Elicit versus the 17 in the published umbrella review. These results highlight Elicit’s potential to support systematic reviews with high precision. Given these developments, rigorously governed AI-assisted rapid review pipelines are becoming increasingly relevant to routine service decision-making.

The present AI-assisted rapid review aims to map the current landscape of PROM use in older adults with T2DM. We focus on instruments assessing psychological, psychosocial, and mental health dimensions in clinical practice between 2015 and 2025, examining which PROMs are used, in what settings, and for what purposes, as well as the factors that facilitate or hinder their implementation. A further objective is to explore how these tools are applied to design patient-centered care pathways and to inform organizational and clinical decision-making. Given the limited number of studies explicitly focused on older adults, our approach adopted a broad inclusion of adult T2DM samples, identifying where findings were directly or indirectly applicable to geriatric care. This choice reflects the pragmatic nature of rapid reviews, which aim to map evidence patterns rather than produce age-restricted estimates.

## 2. Methods

We conducted a rapid review with the support of Elicit, an AI-assisted workflow tool designed to streamline literature retrieval, screening, and data extraction [16]. While Elicit can optionally generate short machine summaries and a concise “synthetic report” to assist users, these functions were not used in this study. All narrative interpretation and synthesis were performed manually by the authors under continuous human supervision. In February 2025, Elicit introduced a “Systematic reviews” workflow that guides researchers through key steps—formulating a research question, identifying relevant literature, suggesting inclusion criteria, extracting data, and producing a synthetic report. To our knowledge, no published paper has yet explicitly used or evaluated this workflow; here, we applied it as an organizational framework but deliberately excluded its automated report from our analysis. Elicit was selected to facilitate literature retrieval and organization under human supervision, consistent with rapid-review guidance.

This review followed the Preferred Reporting Items for Systematic Reviews and Meta-Analyses (PRISMA) 2020 principles where applicable, but no formal protocol registration (e.g., PROSPERO) was performed, as it was designed as an exploratory, AI-assisted rapid evidence mapping. All inclusion and exclusion criteria were pre-specified within the Elicit workflow and consistently applied.

Three authors (MPF, RM, ML) formulated the following research question: “What is the current evidence on the use of Patient-Reported Outcome Measures (PROMs), including self-report questionnaires, in older adults with type 2 diabetes? Specifically, how effectively do PROMs capture psychological, psychosocial, and mental health aspects—such as depression, psychological distress, quality of life, and treatment satisfaction—in real-world clinical practice or routine care settings? Furthermore, how are PROMs being utilized to support risk stratification and to guide personalized care pathways within this population?”.

The query was entered into Elicit with a preliminary filter to include only studies published between 2015 and 2025. Elicit initially retrieved 499 potentially relevant articles indexed in Semantic Scholar, the database on which the tool currently relies. Semantic Scholar is a free AI-based academic search engine developed by the Allen Institute for AI, covering over 200 million publications across scientific fields but not as comprehensive as broader aggregators such as Google Scholar or OpenAlex, and more inclusive but less curated than PubMed or Scopus.

Because Elicit currently operates exclusively on the Semantic Scholar corpus, this review reflects the scope and indexing coverage of that database. While this approach allowed for efficient AI-assisted retrieval and screening, it inherently limited comprehensiveness relative to multi-database searches (e.g., PubMed, Scopus, PsycINFO). To partially address this, manual searches and metadata screening were performed to identify potentially missing studies. To further enhance completeness, five additional recent articles (2024–2025) not yet indexed were manually retrieved and added to the corpus [3,18,19,20,21], as they were considered particularly relevant given their novelty in addressing the research question. Future iterations of this workflow could incorporate multi-database searches (e.g., PubMed, Scopus, PsycINFO) once Elicit or similar tools enable direct multi-source integration.

Elicit generated a preliminary set of screening criteria by analyzing a random sample of 100 sources. These criteria were reviewed and refined by the research team to ensure alignment with study objectives. The final eligibility criteria were (1) inclusion of patients with T2DM; (2) use of validated PROMs or validated self-report questionnaires; (3) assessment of PROM implementation in clinical or real-world contexts, beyond instrument development or validation; and (4) whenever possible, inclusion of older or elderly adults (typically aged ≥65 years) or sub-analyses explicitly referring to age.

Because few studies reported disaggregated data by age, all adult studies meeting the above criteria were retained, and those explicitly focused on older adults were highlighted during extraction. This pragmatic decision reflects the exploratory purpose of the review and the limited availability of strictly geriatric evidence.

The criteria were then applied to the complete set of 504 sources. For each paper, Elicit provided color-graded recommendations (“green”, “yellow”, “red”) for each criterion, together with a narrative justification and a cumulative score from 1 to 5. We verified the reliability of these scores in a subset of papers and decided to include studies with a cumulative score strictly greater than 3.3. Screening reliability was ensured through independent verification by two reviewers, who jointly resolved borderline cases through discussion. The 3.3 inclusion threshold was chosen after pilot testing on a random subset and confirmed to produce stable inclusion patterns. Given the rapid-review framework, no formal inter-rater statistics or sensitivity analysis were conducted, consistent with current practice in rapid evidence syntheses where efficiency and transparency are prioritized over exhaustive validation.

Based on our analysis of papers with values close to the threshold, we manually overrode Elicit’s recommendation in a few cases: three papers with lower scores were included [18,20,22], while one with a higher score was excluded for lack of pertinence to the study aim [23].

At the end of this process, 199 papers were included and 305 excluded. After removing duplicates and records not meeting eligibility criteria, 167 studies remained for inclusion. To ensure no relevant articles had been missed, the metadata (title, authors, year, journal) of excluded papers were manually skimmed. All selection decisions were made collaboratively by the research team, and discrepancies were resolved through discussion.

From the 199 screened-in papers, Elicit randomly selected 10 as a pilot set for initial review. The tool automatically suggested data extraction columns based on our research question, which were then checked and refined by the research team. The final set of variables extracted included study design, PROMs used, participant demographics, key psychological and psychosocial findings, measurement properties and validation, mode of PROM administration, key results, setting, and overall study summary.

Data extraction on the pilot set was examined in detail, including the supporting text segments provided. Once satisfied with the performance of the extraction, the process was run on the full set of 199 papers.

From these, the following were excluded: duplicates (*n* = 13), PhD theses (*n* = 3), studies not meeting the diabetes inclusion criterion (*n* = 6), collections of abstracts without data (*n* = 3), one editorial with no data available (*n* = 1), and qualitative studies (*n* = 6).

The final set comprised 167 papers [2,5,18,20,21,22,24,25,26,27,28,29,30,31,32,33,34,35,36,37,38,39,40,41,42,43,44,45,46,47,48,49,50,51,52,53,54,55,56,57,58,59,60,61,62,63,64,65,66,67,68,69,70,71,72,73,74,75,76,77,78,79,80,81,82,83,84,85,86,87,88,89,90,91,92,93,94,95,96,97,98,99,100,101,102,103,104,105,106,107,108,109,110,111,112,113,114,115,116,117,118,119,120,121,122,123,124,125,126,127,128,129,130,131,132,133,134,135,136,137,138,139,140,141,142,143,144,145,146,147,148,149,150,151,152,153,154,155,156,157,158,159,160,161,162,163,164,165,166,167,168,169,170,171,172,173,174,175,176,177,178,179,180,181,182,183,184]. Among these, approximately one quarter explicitly referred to “older” or “elderly” adults [31,36,49,50,51,56,61,62,64,68,74,75,77,79,83,84,87,90,102,103,106,108,132,137,141,145,147,148,150,153,156,157,160,168,169], while the remainder addressed broader adult populations. Studies were therefore classified as directly or indirectly relevant to older adults to contextualize the findings reported in the Results and Discussion sections. Full texts were obtained for 112 papers (with the support of Elicit browser extensions enabling access to the University of Bologna digital library), while for the remaining papers only titles and abstracts were available. For these records, data extraction was limited to variables explicitly reported in the abstract and verified independently by two reviewers. No interpretive coding was applied, and these studies contributed only to the descriptive mapping rather than to analytic synthesis in order to minimize potential misclassification.

**Figure 1:**
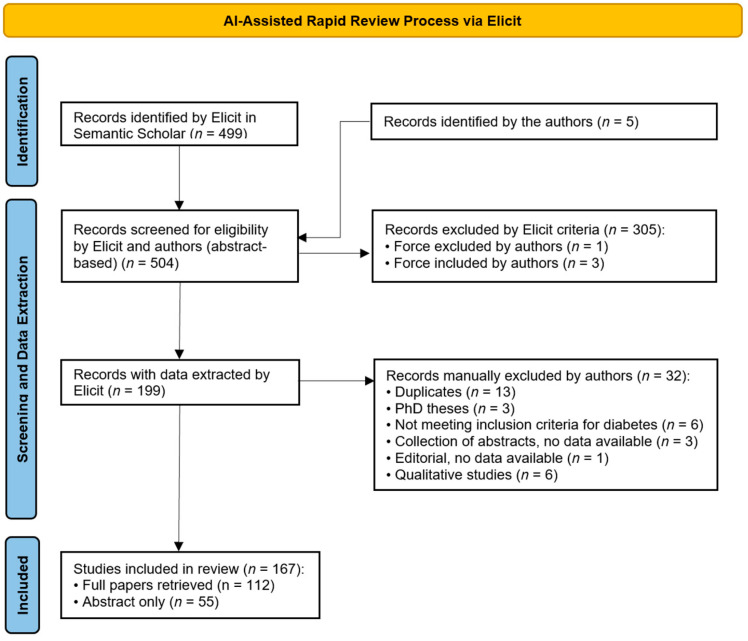
PRISMA Flow Diagram Illustrating the AI-Assisted Rapid Review Process via Elicit.

Figure 1 shows the PRISMA flow diagram of this process. The flow diagram is PRISMA-like rather than fully PRISMA-compliant, as the study represents an AI-assisted rapid evidence mapping rather than a full systematic review. The final data extraction table, covering all columns for the 167 included papers, is provided in the Supplementary Materials (Table S1). For transparency, the Supplementary Materials also include the text excerpts and reasoning trails provided by Elicit during data extraction, allowing readers to trace how suggested information was generated and verified.

Given the rapid-review framework and the AI-assisted workflow, no formal critical appraisal or certainty grading (e.g., GRADE [Grading of Recommendations, Assessment, Development and Evaluation], ROBIS [Risk of Bias in Systematic Reviews]) was undertaken. Instead, evidence strength was inferred qualitatively based on study design, sample size, and consistency of findings across studies. All conclusions were phrased descriptively to reflect this level of evidence.

## 3. Results

The dataset comprised 167 papers published between 2015 and 2025. Most were cross-sectional surveys or systematic reviews, with fewer observational cohorts and randomized controlled trials. Mean sample sizes varied widely, and many studies included adults across the lifespan; approximately one-quarter explicitly described participants as “older” or “elderly”. For clarity, studies were therefore classified as directly relevant (explicitly focused on older or elderly adults) or indirectly relevant (adult samples without specific age stratification). Given the exploratory scope of the review, results integrate both direct and indirect evidence. Abstract-only studies contributed exclusively to the descriptive mapping of PROM use and were not considered in analytic interpretations. Unless otherwise specified, the results reported below reflect patterns across all adult T2DM populations, with comments on age-specific evidence where available.

The following subsections synthesize both direct and indirect evidence on key psychological and psychosocial domains.

Text mining of the extracted fields showed that the psychological and psychosocial outcomes most frequently addressed were diabetes distress, depression, QoL, treatment satisfaction, and anxiety. Commonly used PROMs included the PAID scale [9], Diabetes Distress Scale (DDS) [185], PHQ-9 [8], WHO-5 [6,7], EuroQol-5D (EQ-5D) [186], Short Form-36 (SF-36) [187,188], Diabetes Treatment Satisfaction Questionnaire (DTSQ) [189], Hospital Anxiety and Depression Scale (HADS) [190], and Geriatric Depression Scale (GDS) [191] (Table 1).

### 3.1. Psychological and Psychosocial Outcomes Captured by PROMs

#### 3.1.1. Diabetes Distress

Diabetes distress is defined as the emotional burden and stress associated with the demands of living with diabetes. It is highly prevalent among individuals with T2DM and consistently linked to poorer self-management, reduced treatment adherence, and worse glycemic outcomes [78,85]. Older adults may be particularly vulnerable, as they often face compounding challenges such as longer disease duration, declining physical capacity, and increased fear of complications, which amplify emotional strain and hinder effective self-care [75,138]. This underscores the need for targeted interventions to mitigate distress and improve outcomes in this population. However, relatively few studies explicitly analyzed distress patterns by age or focused exclusively on older cohorts, so these considerations are based on indirect evidence supported by consistent contextual factors.

Evidence suggests that interventions specifically designed to address diabetes distress can be effective. A recent meta-analysis found that programs—especially those delivered in group formats, incorporating cognitive-behavioral strategies, and supported by digital tools—significantly reduced distress in adults with T2DM, though long-term effects remain uncertain [159]. Digital health approaches, such as mobile health platforms, have also shown promise in reducing distress and supporting self-management, offering scalable options that may be particularly beneficial for older adults with limited access to in-person services [43].

Validated PROMs are commonly used to assess diabetes distress and evaluate intervention effectiveness. The PAID scale, a 20-item questionnaire [9], and its shorter validated versions, PAID-5 and PAID-1 [192], capture the negative emotions related to living with diabetes. The DDS [185], consisting of 17 items, measures emotional burden, physician- and regimen-related distress, and interpersonal distress. Both instruments demonstrate strong psychometric properties and sensitivity to change, making them valuable in both research and clinical contexts. In most studies, PAID or DDS scores have been employed to quantify baseline distress and monitor intervention outcomes [193], although their routine use in clinical practice remains limited. Importantly, large-scale evidence such as the Swedish National Diabetes Register [111] has shown the utility of incorporating distress measures alongside clinical risk factors (e.g., glycated hemoglobin [HbA1c], blood pressure, low-density lipoprotein [LDL] cholesterol). This enables the identification of patient subgroups in need of more tailored support, based on both psychosocial and biomedical profiles.

#### 3.1.2. Depression

Depression is a prevalent comorbidity in T2DM, significantly impacting treatment adherence [194] as well as the risk of complications and mortality [195,196]. Epidemiological studies indicate that the incidence of major depressive disorder among individuals with T2DM is substantial, with higher depressive symptom scores linked to increased diabetes distress and psychosocial stress [80]. The use of PROMs in clinical practice, although still underutilized, has demonstrated potential to improve both depressive symptoms and metabolic outcomes [63]. Digital health innovations, such as personalized care plans delivered via mobile applications, have further enhanced the feasibility and accessibility of monitoring mental health and self-management in diabetes [70].

Systematic evaluation of depression is particularly important because untreated depressive symptoms are associated with higher mortality, reduced adherence, poorer disease management, and lower QoL [163]. Guidelines recommend the use of standardized screening tools for depression in people aged more than 65 years [137]. Several instruments have been validated for use in older adults, including the GDS, Center for Epidemiologic Studies Depression Scale (CES-D) [197], Beck Depression Inventory (BDI) [198], WHO-5, and PHQ-9. Despite their established validity, the integration of these screening tools into routine clinical practice remains limited, and few studies have employed repeated measures to monitor changes in depressive symptoms following interventions [63]. Notably, only a minority of included studies explicitly targeted participants aged ≥65 years, highlighting a gap between guideline recommendations for geriatric screening and current research practice.

#### 3.1.3. Anxiety

Only a few studies assessed anxiety in T2DM, despite its strong impact on self-care behaviors and overall disease management. Among the available tools, the HADS [190] is the most widely used, as it allows a quick and reliable screening of both anxiety and depression. In fact, in South Africa Ramkisson et al. reported 32% of patients with mild-to-severe anxiety on HADS [40], while in Romania Pah et al. [164] found a much higher prevalence of 62.2%, with anxiety being more frequent in patients with macrovascular complications. These findings highlight not only the relevance of anxiety in T2DM but also the need for greater routine attention to its evaluation.

#### 3.1.4. Quality of Life (QoL) and Well-Being

In older adults with T2DM, quality of life can be reduced due to the burden of disease, complications, treatments, and age-related vulnerabilities such as frailty and cognitive decline. Assessing both general and diabetes-specific QoL can guide patient-centered care and shared decision-making, but system-level changes are needed to integrate QoL measurement into routine practice [157].

Among the studies explicitly addressing older adults, generic instruments such as EQ-5D and SF-36 predominated over disease-specific tools like ADDQoL or DQoL, suggesting that psychometric adaptation for geriatric populations remains limited. Generic instruments such as the EQ-5D [186] and SF-36 [187,188] are widely used and allow comparisons across populations, while diabetes-specific measures include the Audit of Diabetes-Dependent Quality of Life (ADDQoL) [199], Diabetes Quality of Life (DQoL) [200] questionnaires, and the WHO-5 (Table 1).

A recent systematic review [143], including 40 studies, identified the SF-12 and the SF-36 as the commonly used questionnaires to evaluate QoL in people with T2DM. In our dataset, EQ-5D, SF-36 and WHO-5 were the most frequently used generic QoL measures.

According to another systematic review [112], in research, PROMs evaluating QoL are widely used in clinical trials, allowing investigators to evaluate how treatments affect not only biomedical markers but also daily functioning and overall psychological well-being. In clinical practice, they provide a structured way to understand a patient’s baseline status, track changes over time, and support more personalized, shared decision-making in diabetes care.

#### 3.1.5. Treatment Satisfaction

Treatment satisfaction is a distinct patient-reported outcome that evaluates individuals’ perceptions of their diabetes therapy [201]. Evaluating treatment satisfaction provides insights beyond HbA1c, reflecting patients’ experiences with therapy. Higher satisfaction is associated with better adherence, greater self-efficacy, and lower risk of dropout [174]. The DTSQ was the most commonly used instrument in the included studies (Table 1).

In the PANORAMA study [128], the DTSQ was administered alongside the ADDQoL. Findings showed that depression, weight gain, and complex hypoglycemic regimens were associated with lower satisfaction scores. Interestingly, patients rated their satisfaction higher than their physicians did, suggesting that clinicians may underestimate treatment burden. These results highlight the value of PROMs in identifying mismatches between patient and physician perspectives.

#### 3.1.6. Self-Efficacy and Self-Management

Self-efficacy, a psychological construct derived from Social Cognitive Theory, refers to a person’s belief in their ability to carry out actions needed to manage specific situations [202]. Unlike broader ideas such as self-esteem or self-confidence, it is task- and context-specific. In diabetes care, the American Diabetes Association highlights the importance of considering patients’ treatment burden and their confidence in managing daily self-care [137]. High levels of self-efficacy are strongly linked to better self-management of chronic conditions, making it a key target for healthcare providers [203]. In our dataset, some of the included studies assessed both self-efficacy and self-management activities [44,47,49,50,58,62,64,73,74,84,86,88,99,106,135,136,150,158,170,181,184]. Diabetes-specific questionnaires to evaluate self-efficacy were the Diabetes Management Self-Efficacy Scale (DMSES) [204] and the Diabetes Self-Efficacy Scale (DSES) [205]. To measure levels of self-management activities, the most used scale was the Summary of Diabetes Self-Care Activities (SDSCA) [206]. Higher self-efficacy scores were consistently associated with better glycemic control and treatment adherence, consistent with evidence that self-efficacy mediates the relationship between psychological aspects, self-care behaviors, and HbA1c levels.

Given the strong influence of psychological factors on self-care behaviors, more research is needed to evaluate self-efficacy and empowerment interventions in older adults with T2DM.

### 3.2. Using PROMs for Risk Stratification and Personalized Care

PROM administration was predominantly through self-report paper questionnaires, with only a minority of studies adopting digital formats [24,33,34,36,73,81,82,98,100,115,127,134,138,175] or repeated measures [60,70,74,115,175]. Settings were mainly outpatient clinics or community-dwelling older adults, while primary care and general practice were underrepresented. Several studies report that PROMs can stratify patients by distress, depression, or quality of life, identifying those at higher risk for poor outcomes [18,39,95]. PROMs are associated with demographic and clinical risk factors and this can be used to identify fragile people. PROMs can be used to trigger tailored interventions, referrals, or care planning [63,70]. Moreover, studies show that PROM-guided interventions can improve mental health outcomes, self-management, and quality of life [49,113,117,177]. Nonetheless, real-world integration into care pathways is still inconsistent, PROMs in the dataset were used primarily for research or one-off surveys rather than as routine clinical tools.

### 3.3. Implementation Challenges and Solutions

Integration of PROMs into routine care is inconsistent, with barriers including time, workflow disruption, lack of provider training, and cultural inappropriateness of some PROMs [42,63]. Facilitators for the implementation of PROMs in clinical practice include digital administration, brief and validated tools (e.g., PAID-5, WHO-5) and integration with care planning software [70,82,122]. However, digital literacy and access represent additional barriers in older populations. Community and patient engagement in tool development could enhance acceptability [42]. Implementation requires investment in digital infrastructure, training for the healthcare professionals, and workflow redesign. Addressing barriers and leveraging facilitators is essential for successful PROM implementation. Tailoring tools and processes to local healthcare organizations, patient populations, and resource availability is critical. While barriers such as low digital literacy and accessibility disproportionately affect older adults, few studies offered age-specific implementation analyses. This highlights an important direction for future research and policy.

## 4. Discussion

This review illustrates the feasibility of applying an AI-assisted rapid review methodology to address a focused clinical and organizational question concerning adults with T2DM, with specific attention to older populations. Beyond the novelty of the approach [207,208], our analysis highlights consistent patterns in the use of PROMs and provides insights into both their value and their current limitations in practice.

A key limitation of the evidence synthesized is that only about one quarter of the included studies explicitly involved older or elderly adults, while the remainder referred to broader adult populations. As a result, some of the conclusions drawn here reflect patterns applicable to adults in general rather than exclusively to geriatric care. Where age-specific data were available, findings were reported separately, but these instances were relatively few. Therefore, the present review should be interpreted as providing an exploratory mapping of PROM use in adult T2DM populations, highlighting insights that are directly or indirectly transferable to older adults.

The principal findings confirm that PROMs are effective in capturing psychosocial and psychological burdens that often remain invisible in routine clinical encounters. Disease-specific instruments such as PAID, DDS, ADDQoL, and DTSQ showed greater sensitivity to diabetes-related concerns than generic tools (e.g., EQ-5D, SF-36, WHO-5). Depression and distress measures consistently revealed high levels of psychological burden, while QoL and treatment satisfaction instruments captured the tangible impact of diabetes on everyday functioning. However, implementation in routine care is still limited, hindered by heterogeneity of instruments, insufficient validation in older populations, lack of cultural adaptation, and uncertainty on how to translate scores into actionable care.

An important limitation is the lack of PROMs specifically designed to assess the impact of diabetes technologies [120]. As diabetes management increasingly relies on digital tools—such as continuous glucose monitoring [31], insulin pumps, and app-based decision aids—it becomes crucial to understand how patients experience and adapt to these technologies. PROMs that capture technology acceptance, satisfaction, perceived burden, and usability are still in early stages of development, with limited application in research and almost no routine use in clinical care [183]. This gap is particularly relevant given the rapid pace of innovation in diabetes care, which is reshaping not only clinical pathways but also how individuals live with and relate to their condition, indicating that existing PROMs may need to be adapted or expanded to capture these changing dynamics. In this context, it becomes essential to extend PROM development to domains such as technology acceptance, digital literacy, and perceived usability. These aspects are particularly relevant for older adults, who may face additional barriers to engaging with diabetes technologies yet could benefit greatly from tools that enhance autonomy and self-management. Incorporating technology-related dimensions into PROM frameworks would improve their relevance for contemporary diabetes care pathways.

From a methodological standpoint, this study demonstrates the potential of semi-automated, AI-supported pipelines to generate timely, evidence-informed syntheses. In practice, the AI-assisted workflow allowed completion of all review stages—literature retrieval, screening, and data extraction—within weeks while maintaining transparent, reproducible documentation at each step. Using Elicit allowed us to screen a large body of literature efficiently and transparently, while still requiring human oversight for refinement, judgment, and contextualization. This dual track—automation for efficiency, expert review for quality—offers a model for how rapid evidence products could be produced at scale, especially when clinical or organizational decisions need to be made under time constraints.

The review was not prospectively registered, as no formal protocol was developed a priori. However, all methods and eligibility criteria were pre-specified and transparently documented within the Elicit workflow, consistent with the exploratory nature of AI-assisted rapid reviews.

Although the synthesis was primarily descriptive and did not include formal critical appraisal or certainty grading, this approach is consistent with established rapid-review methodologies, which prioritize timeliness and scope over exhaustive quality assessment. To maintain transparency, evidence strength was interpreted qualitatively based on study design, sample size, and consistency across findings, in line with the pragmatic aims of rapid evidence mapping rather than formal graded recommendations.

At the same time, reliance on a single bibliographic source (Semantic Scholar) and the lack of independent validation highlight limitations that call for cautious interpretation and further external testing. This limitation stems from the current configuration of Elicit, which at the time of the review operated solely on the Semantic Scholar index. Although mitigation measures such as manual additions and metadata screening were applied, future replications should be expanded to multiple databases to enhance coverage and reproducibility. Another limitation was that data extraction for a subset of papers relied only on abstracts, potentially reducing detail and depth. To mitigate this risk, all abstract-only records were double-checked for key variables by two reviewers, and data were extracted only for information explicitly reported in the source text. No inferential coding or extrapolation was performed. As a result, these records contributed only to descriptive mapping rather than to analytic synthesis, minimizing the potential for misclassification bias.

Clinically, the evidence points to multiple potential roles for PROMs: identifying high-risk patients, tailoring interventions, monitoring outcomes, and supporting shared decision-making. Integration of patient-reported data with clinical risk factors, as illustrated in the Swedish National Diabetes Register [111], suggests a pathway toward more comprehensive risk stratification models. Such integration could improve the targeting of interventions, particularly in older adults with multimorbidity and frailty, where biomedical indicators alone are insufficient.

For health systems, wider use of PROMs could contribute to improving adherence to organizational and clinical guidelines by making patient perspectives visible in routine practice. Equally important, their use requires adequate training of healthcare professionals to interpret scores, address sensitive psychological issues, and act upon findings in a timely manner. Without such capacity building, PROMs risk remaining research tools rather than instruments embedded in everyday care.

Digitizing PROMs can streamline their collection and integration into routine diabetes care, enhancing patient monitoring and engagement. This approach supports more personalized treatment decisions and may contribute to improved outcomes [33].

Looking forward, digital and hybrid approaches—ranging from mobile applications to integration into electronic health records—hold promise for expanding the reach and sustainability of PROM collection. Future work should also prioritize validation of PROMs in older adults, exploration of longitudinal trajectories, and development of predictive models that combine biomedical and patient-reported data. These steps are essential to move from descriptive use of PROMs toward their systematic integration in personalized care pathways for people with T2DM, ultimately bridging the gap between research and routine practice. In particular, validation and adaptation of PROMs for older adults—with attention to cognitive accessibility, comorbidity, and functional status—remain key research priorities.

## 5. Conclusions

This AI-assisted rapid review mapped the use of PROMs in adults with T2DM between 2015 and 2025, with a specific focus on older populations. Evidence suggests that PROMs effectively capture psychosocial and psychological dimensions often missed in routine encounters, with disease-specific instruments offering more targeted insights than generic tools.

However, only a subset of the included studies explicitly involved older or elderly participants, and conclusions for this population should therefore be interpreted with caution. Broader adoption of PROMs in geriatric care will require targeted validation, simplified tools, and integration into workflows that account for age-related functional and cognitive differences. Moreover, the rapid digitalization of diabetes management underscores the need for PROMs that capture technology-related experiences, including usability, satisfaction, and perceived burden. Developing and validating such instruments will be essential to ensure that patient-reported data remain aligned with evolving models of digital and hybrid care.

From a methodological perspective, this review illustrates how semi-automated pipelines such as Elicit can deliver timely, evidence-informed syntheses when coupled with expert oversight. Future research should prioritize longitudinal validation, age-stratified analyses, and integration of PROMs with biomedical data to move from exploratory mapping toward personalized, age-sensitive care pathways.

## Figures and Tables

**Table 1:** Patient-Reported Outcome Measures (PROMs) Most Frequently Applied in Adults with Type 2 Diabetes Mellitus (T2DM), including Studies Involving Older Populations: Findings from an AI-Assisted Rapid Review.

PROM	Psychological Domain	Description	References
Problem Areas in Diabetes (PAID)	Diabetes-related emotional distress; fear of complications; emotional burden from diabetes self-management	20-item scale (short versions: PAID-5, PAID-1). Widely used; sensitive to change; distinguishes distress from depression.	[2,5,18,21,26,28,39,42,46,53,67,68,71,76,77,80,85,86,88,89,92,95,99,114,125,129,131,136,142,150,159,167,172,178,179]
Diabetes Distress Scale (DDS)	Emotional burden; physician-related distress; regimen distress, interpersonal distress	17-item scale; includes four subdomains.	[2,22,25,43,70,71,79,91,92,104,112,113,130,138,145,146,151,158,159,163,166]
Patient Health Questionnaire-9 (PHQ-9)	Depressive symptoms and severity	Brief screening tool validated in older adults; positive screens require diagnostic confirmation.	[5,18,21,28,43,46,63,70,74,80,89,99,104,106,112,129,134,137,143,146,151,158,163,166,175,176]
World Health Organization-5 Well-Being Index (WHO-5)	Psychological well-being	Short tool for depression screening; validated in T2DM; widely used in QoL research.	[5,18,21,26,28,40,53,58,59,63,80,86,89,98,142,143,184]
EuroQol-5D (EQ-5D)	Generic health status (mobility, self-care, usual activities, pain/discomfort, anxiety/depression)	Generic QoL instrument; allows comparison across populations; not specific to diabetes.	[24,31,58,70,71,73,97,99,109,114,116,119,125,128,134,136,143,170,173]
Short Form-36 (SF-36/SF-12)	Physical and mental health domains (physical functioning, role limitations, pain, general health, vitality, social functioning, emotional well-being, mental health)	Generic QoL instruments; widely used; normative data available.	[2,21,31,49,59,64,71,99,106,112,119,141,143,145,151,165]
Diabetes Treatment Satisfaction Questionnaire (DTSQ)	Treatment satisfaction; convenience; flexibility; perceived hyper/hypoglycemia	Validated in multiple languages; positive association with adherence; conceptually distinct from QoL.	[59,71,73,99,100,114,119,128,141,165,173,174]
Hospital Anxiety and Depression Scale (HADS)	Anxiety and depression (two subscales)	Brief screening tool; detects comorbid anxiety; less used in older adults with T2DM.	[40,43,83,99,134,164,170]
Audit of Diabetes-Dependent Quality of Life (ADDQoL)	Diabetes-related QoL across multiple domains	Disease-specific QoL instrument; generates negative impact scores	[59,62,78,88,99,112,113,119,123,128,134,143,173,178]
Geriatric Depression Scale (GDS)	Depression in older adults	Specifically designed for older adults; available in 15- and 30-item versions; recommended for routine screening	[50,56,132]

## Data Availability

Data sharing is not applicable to this article.

## Supplementary Materials

The following supporting information can be downloaded at: https://www.mdpi.com/article/10.3390/healthcare13222840/s1, Table S1: Elicit’s automated Data Extraction for the 167 Included Studies: Study Design, Patient-Reported Outcome Measures (PROMs), Populations, Key Findings, and Implementation Details.

## Abbreviations

The following abbreviations are used in this manuscript:

T2DM	Type 2 diabetes mellitus
QoL	Quality of life
PROMs	Patient-Reported Outcome Measures
ICHOM	International Consortium for Health Outcomes Measurement
WHO-5	World Health Organization–Five Well-Being Index
PHQ-9	Patient Health Questionnaire-9
PAID	Problem Areas in Diabetes
AI	Artificial intelligence
PRISMA	Preferred Reporting Items for Systematic Reviews and Meta-Analyses
GRADE	Grading of Recommendations, Assessment, Development and Evaluation
ROBIS	Risk of Bias in Systematic Reviews
DDS	Diabetes Distress Scale
EQ-5D	EuroQol-5D
SF-36	Short Form-36
DTSQ	Diabetes Treatment Satisfaction Questionnaire
HADS	Hospital Anxiety and Depression Scale
GDS	Geriatric Depression Scale
HbA1c	Glycated hemoglobin
LDL	Low-density lipoprotein
CES-D	Center for Epidemiologic Studies Depression Scale
BDI	Beck Depression Inventory
ADDQoL	Audit of Diabetes-Dependent Quality of Life
DQoL	Diabetes Quality of Life
DMSES	Diabetes Management Self-Efficacy Scale
DSES	Diabetes Self-Efficacy Scale
SDSCA	Summary of Diabetes Self-Care Activities
